# Validation of a non-invasive pressure–time index of the inspiratory muscles in spontaneously breathing newborn infants

**DOI:** 10.1007/s10877-022-00882-6

**Published:** 2022-06-08

**Authors:** Theodore Dassios, Aggeliki Vervenioti, Sotirios Tzifas, Sotirios Fouzas, Gabriel Dimitriou

**Affiliations:** 1 Department of Women and Children’s Health, King’s College London, London, UK; 2 Neonatal Intensive Care Unit, Department of Paediatrics, University of Patras, Patras, Greece; 3 Paediatric Pulmonology Unit, Department of Paediatrics, University of Patras, Patras, Greece; 4 Neonatal Intensive Care Centre, King’s College Hospital, 4th Floor Golden Jubilee Wing, Denmark Hill, London, SE5 9RS UK

**Keywords:** Neonates, Respiratory muscle function, Pressure time index of the diaphragm, Pressure time index of the respiratory muscles

## Abstract

To validate the pressure–time index of the inspiratory muscles as a non-invasive index of inspiratory muscle function in spontaneously breathing infants by comparing it against the gold-standard pressure–time index of the diaphragm. Prospective observational cohort study of consecutive infants breathing unsupported in room air in a tertiary neonatal intensive care unit, studied prior to discharge from neonatal care. The invasive pressure–time index of the diaphragm was calculated using a transdiaphragmatic dual-pressure catheter that measured transdiaphragmatic pressure by subtraction of the oesophageal from the gastric pressure. The non-invasive pressure–time index of the inspiratory muscles was calculated using pressure measurements at the level of the mouth via a differential pressure transducer connected to a face mask. Both indices were calculated as the product of the ratio of the mean inspiratory pressure divided by the maximum inspiratory pressure and the ratio of the inspiratory time divided by the total time of a respiratory cycle. One hundred and thirty infants (79 male) were included with a mean (SD) gestational age of 35.2 (3.2) weeks, studied at a median (IQR) postnatal age of 9 (6–20) days. The mean (SD) pressure–time index of the diaphragm was 0.063 (0.019) and the mean (SD) pressure–time index of the inspiratory muscles was 0.065 (0.023). The correlation coefficient for the two indices was 0.509 (p < 0.001). The mean (SD) absolute difference between the pressure–time index of the inspiratory muscles and pressure–time index of the diaphragm was 0.002 (0.021). In convalescent infants, the non-invasive pressure–time index of the inspiratory muscles had a moderate degree of correlation with the invasively derived pressure time index of the diaphragm measured with a transdiaphragmatic catheter.

## Introduction

Impaired respiratory muscle function may lead to progressive muscle fatigue, which could result in an inability to maintain adequate alveolar ventilation, and subsequent respiratory failure [1]. Infants, especially those born prematurely, are prone to diaphragmatic dysfunction due to limited reserves and a limited capacity to generate force and avoid fatigue [2]. Assessment of respiratory muscle function may help to identify newborn infants that may be susceptible to respiratory muscle fatigue. One clinical application of respiratory muscle assessment in newborns is that impaired respiratory muscle function could potentially predict extubation failure in ventilated infants [3–5].

Methods to assess the respiratory muscles in the newborn include electromyography, volitional maximum respiratory pressures and assessment for thoraco-abdominal asynchrony [2]. Composite indices can also be used, one of which is the diaphragmatic pressure–time index (PTIdi), which is the product of the ratio of the mean to the maximum transdiaphragmatic pressure and the inspiratory duty cycle [6]. The PTIdi describes the pressure-generating activity of the diaphragm and assesses the balance between the capacity of the diaphragm to generate force and the load imposed upon it. In concept, the longer the inspiratory muscles contract during a breathing cycle and the higher a fraction of their maximum force they use, the less efficient and more fatigue-prone they are. A high PTIdi can assess the load on the respiratory muscles, and predict extubation outcome in children [7]. The determination of the PTIdi, however, is invasive and requires the use of oesophageal and gastric catheters. Alternatively, a non-invasive method of assessing the pressure–time index of the inspiratory muscles (PTImus) has been successfully validated in adults and exhibited a highly-significant correlation with the PTIdi [8]. The PTImus has not been previously measured in spontaneously breathing infants.

Our aim was to compare the PTIdi and PTImus in spontaneously breathing infants and to determine whether the PTImus could provide an alternative method to assess respiratory muscle performance in newborn infants.

## Methods

### Study design and participants

A prospective observational cohort study of newborn infants admitted in the Neonatal Intensive Care Unit of the University Hospital of Patras, Greece, between February 2008 and February 2009 was undertaken. Infants were consecutively enrolled and studied within 48 h prior to discharge. They were included in the study when they were not on any respiratory support and were breathing in room air. Infants were not on sedative agents and did not receive any methylxanthines prior to measurement. Infants with moderate/severe hypoxic ischaemic encephalopathy and major congenital abnormalities were excluded. The study was approved by the Research Ethics Committee of the University Hospital of Patras, Greece and parents gave written informed consent prior to enrolment.

### Assessment of respiratory muscle function

**Fig. 1:**
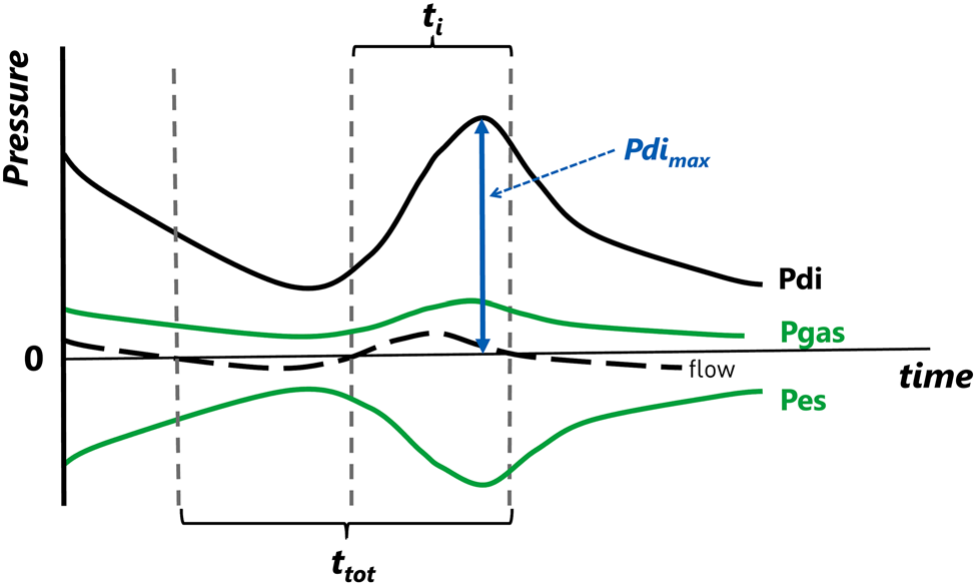
Diagram of pressure versus time demonstrating the calculation of the transdiaphragmatic pressure (Pdi) from the esophageal (Pes) and gastric pressure (Pgas) signals. The phase change of the respiratory flow trace (dashed line) is used to define the inspiratory time (Ti)

**Fig. 2:**
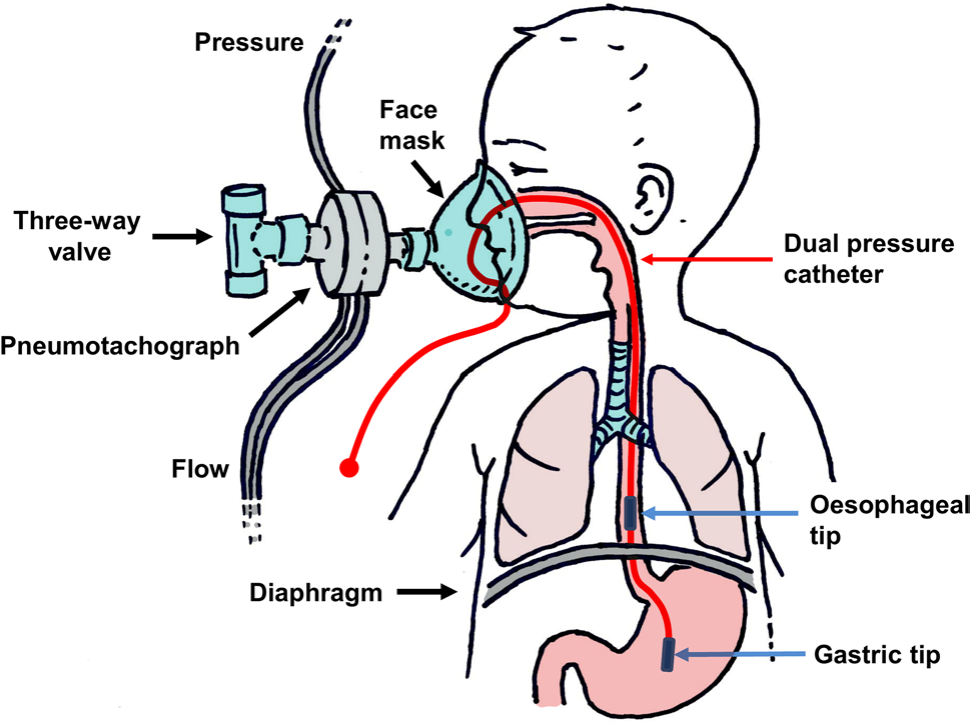
Schematic representation of the experimental setup for the measurement of the PTIdi via the dual pressure catheter and the PTImus via the pressure sensor connected to the pneumotachograph and the face mask. The unidirectional three-way valve that was used to perform the occlusions is also depicted

Respiratory muscle function was assessed with the PTIdi and the PTImus. Higher values of PTIdi and PTImus correspond to impaired respiratory muscle function, reduced capacity of the respiratory muscles and a higher risk of respiratory muscle fatigue under conditions of increased inspiratory load. The airflow signal was used to determine the onset and end of the inspiratory time and the duration of the respiratory cycle (Fig. 1). Airflow was measured by a pneumotachograph (Mercury F10L; GM Instruments, Kilwinning, Scotland, UK) connected to a facemask (total dead space, 4.5 mL) which was held tightly over the infant's nose and mouth to minimise any potential leak (Fig. 2). The pressure and flow signals were recorded and displayed in real time on a computer (Dell Optiplex GX620; Dell Inc, Round Rock, TX), running a Labview application (National Instruments, Austin, TX) with analog to digital sampling at 100 Hz (16-bit NI PCI-6036E, National Instruments).

### Measurement of PTIdi

The transdiaphragmatic pressure (Pdi) was obtained by digital subtraction of the oesophageal (Pes) from the gastric pressure (Pgas) measured using a flexible, silicone-coated catheter (7 French gauge) fitted with two microtransducers (CTO/2-1, Gaeltec Ltd, Dunvegan, Scotland, UK, Fig. 1). The pressure transducers were five centimetres apart, with the distal transducer located 0.3 cm from the tip of the catheter. The correct positioning of the catheter on either side of the diaphragm was checked by comparing Pes to airway pressure during an occluded breath, as previously described [9]. The oesophageal pressure tip of the catheter was positioned in the stomach and then withdrawn until a negative pressure deflection was recorded during inspiration [9]. The airway was occluded at the end of a spontaneous crying effort using a three-way, unidirectional valve attached to the pneumotachograph, which allowed expiration, but not inspiration. The occlusion was maintained for at least four inspiratory efforts. At least three sets of airway occlusions were performed and the maximum Pdi (Pdi_max_) achieved for each subject was recorded [10]. The mean airway pressure (Pdi_mean_) was calculated as the integral of the airway pressure during inspiration, while the infant was quietly breathing, calculated by the computer software [6].

The pressure–time index of the diaphragm (PTIdi), was calculated as:

$$  {\text{PTIdi}}\, = \,\left( {\frac{{{\text{Pdi}}_{{{\text{mean}}}} }}{{{\text{Pdi}}_{{{\text{max}}}} }}} \right)\, \times \,\left( {\frac{{{\text{Ti}}}}{{{\text{Ttot}}}}} \right),  $$

where Ti was the inspiration time and Ttot was the total time for each breath, calculated from the airway flow signal.

### Measurement of PTImus

The PTImus is the non-invasive equivalent of the PTIdi, and can be measured at the level of the mouth without the insertion of a transdiaphragmatic catheter. The pressure generated 100 ms after an occlusion (P_0.1_) was measured while the infant was quietly breathing. At least four airway occlusions were performed and the average P_0.1_ was calculated. The pressures for the calculation of the PTImus (P_0.1_ and Pimax) were measured from a side port on the pneumotachograph which was connected to a differential pressure transducer (Validyne MP 45, range ± 100 cm H_2_O; Validyne Corporation, Northridge, California, USA). The PTImus was calculated as:

$$   {\text{PTImus}}\, = \,\left( {\frac{{{\text{Pi}}_{{{\text{mean}}}} }}{{{\text{Pi}}_{{\max }} }}} \right)\, \times \,\left( {\frac{{{\text{Ti}}}}{{{\text{Ttot}}}}} \right),   $$

where Pi_mean_ was the average airway pressure during inspiration, obtained from the formula Pi_mean_ = 5 × P_0.1_ × Ti [11]. Pi_max_ was the maximum inspiratory airway pressure measured after the airway was occluded at the end of a spontaneous crying effort using the three-way, unidirectional valve as described in the above paragraph (Fig. 2) [6, 9]. The measurements of Pdi_max_ and Pi_max_ were performed simultaneously. At least three sets of airway occlusions were performed and the maximum Pi (Pi_max_) achieved for each subject was recorded [6, 9, 10]. All calculations were automatically performed by the data acquisition software and the PTIdi and PTImus were studied in random order.

### Data from the medical notes

The following data were collected from the medical notes: gestational age (weeks), birth weight (kg), sex, complete course of antenatal steroids, method of delivery, Apgar score at 5 min, surfactant administration and duration of mechanical ventilation (days). The birth weight z-score was calculated using the UK-World Health Organization (WHO) preterm reference chart [12] and the Microsoft Excel add-in LMS Growth (version 2.77; www.healthforchildren.co.uk).

### Statistical analysis

Data were tested for normality using the Shapiro–Wilk and D’Agostino skewness tests. Normally distributed variables were presented as mean and standard deviation (SD) and non-normally distributed data as median and interquartile range. The relationship of PTIdi with PTImus was examined using Pearson’s correlation analysis. Univariate regression analysis was performed to determine the ability of the PTImus to predict the PTIdi. Analysis of agreement between the PTIdi and the PTImus was performed according to Bland and Altman [13]. The relationship of the difference between the PTImus and the PTIdi (PTImus minus PTIdi) with the gestational age, birth weight, postmenstrual age and history of mechanical ventilation was assessed using multivariable regression analysis with the difference between PTImus and PTIdi as the outcome variable. The PTIdi and PTImus were compared in infants with a history of mechanical ventilation and infants without a history of mechanical ventilation using paired t-test. The statistical analysis was performed using SPSS software, version 26.0 (IBM, Armonk, NY, USA).

## Results

One hundred and thirty infants (79 male) were included with a mean (SD) gestational age of 35.2 (3.2) weeks and birth weight of 2.53 (0.82) kg, (Table 1). They were studied at a median (IQR) postnatal age of 9 (6–20) days (Table 1). Sixty-eight infants (52%) were mechanically ventilated during their stay and 63 (48%) received surfactant.

**Table 1:** Characteristics of the included infants

	Mean (standard deviation), median (interquartile range) or *N* (%)	Range
Gestational age (weeks)	35.2 (3.2)	27–40
Birth weight (kg)	2.53 (0.82)	0.90–4.54
Birth weight z-score	− 0.04 (1.03)	− 3.06–2.51
Male sex	79 (61)	N/A
Antenatal steroids	43 (33)	N/A
Caesarian section	95 (73)	N/A
Apgar score at 5 min	9 (8–10)	3–10
Invasive mechanical ventilation	68 (52)	N/A
Duration of invasive ventilation (days)*	3 (2–5)	1–59
Surfactant	63 (48)	N/A
Age at study(days)	9 (6–20)	1–107

*N* = 130

*In ventilated infants only

**Fig. 3:**
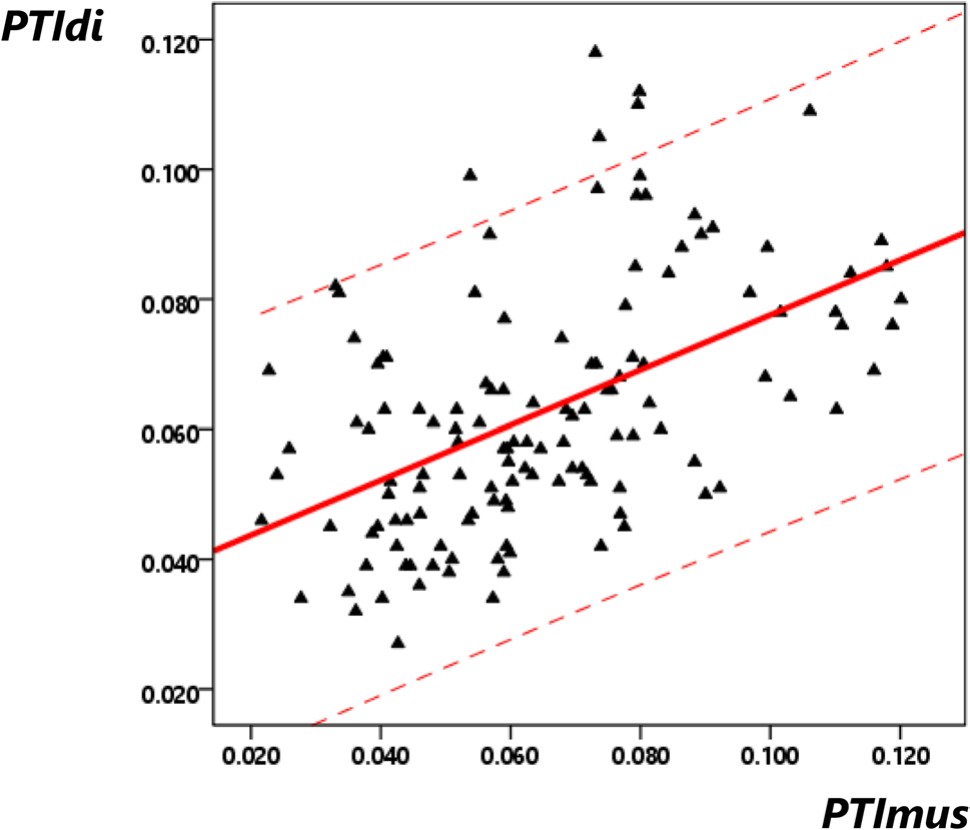
Univariate linear regression analysis of the PTImus to predict the PTIdi. The dashed lines represent the 95% confidence intervals

**Fig. 4:**
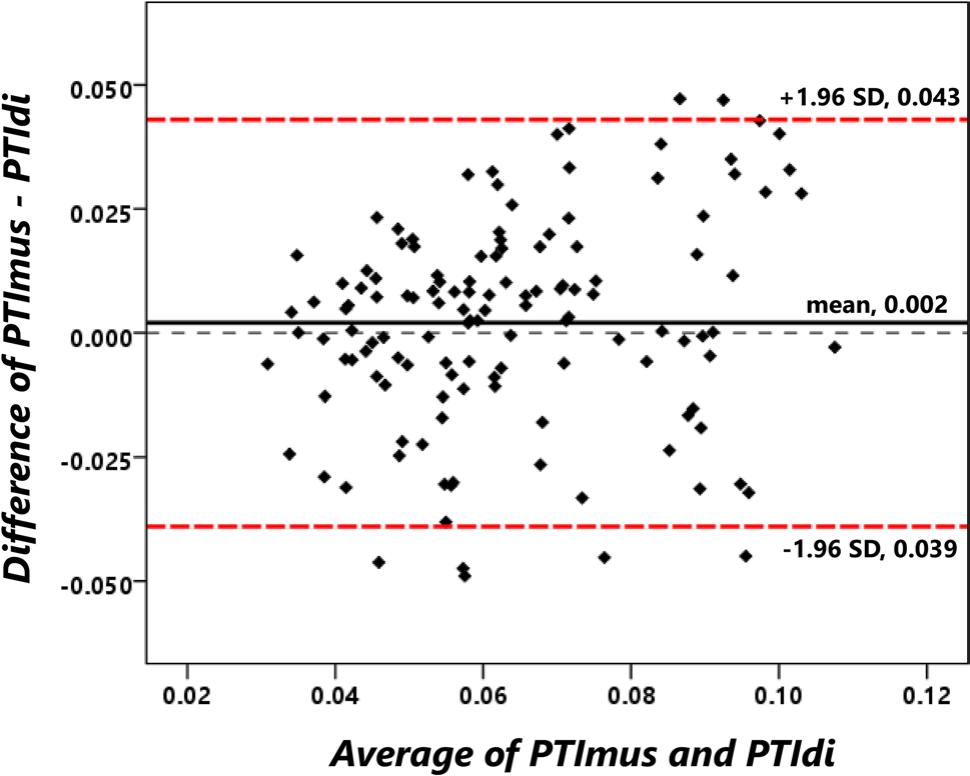
Bland Altman plot of the differences between the PTImus minus PTIdi over the average values

The mean (SD, range) PTIdi was 0.063 (0.019, 0.027–0.118) and the mean (SD, range) PTImus was 0.065 (0.023, 0.022–0.120). The components of the PTIdi and PTImus are presented in Table 2. The two indices were correlated (r = 0.509, p < 0.001) and following univariate regression the PTImus could predict the PTIdi (R^2^ = 0.259, p < 0.001, 95% Confidence Intervals: 0.298–0.549, Fig. 3). The mean (SD) absolute difference between PTImus and PTIdi was 0.002 (0.021). The Bland Altman plot of the difference between the two indices is presented in Fig. 4.

**Table 2:** Components of the PTIdi and PTImus

	Mean (standard deviation)
Inspiratory time (Ti)	0.42 (0.10)
Ti/Ttot	0.42 (0.05)
Maximum transdiaphragmatic pressure (Pdi_max_) (cmH_2_O)	83.5 (14.5)
Maximum inspiratory pressure (Pi_max_) (cmH_2_O)	65.2 (14.6)
Pressure 100 ms after occlusion (P_0.1_) (cmH_2_O)	4.7 (1.5)
Mean transdiaphragmatic pressure (Pdi_mean_) (cmH_2_O)	12.2 (3.4)
Mean airway pressure (Pi_mean_)(cmH_2_O)	9.7 (3.2)
PTIdi	0.063 (0.019)
PTImus	0.065 (0.023)

Following multivariable regression analysis the difference between PTIdi and PTImus was independently associated with the postmenstrual age at study (adjusted p = 0.024, standardised coefficient = − 0.293) but not with the gestational age (adjusted p = 0.580), birth weight (adjusted p = 0.499) and history of mechanical ventilation (adjusted p = 0.882). In infants with a history of mechanical ventilation, the mean (SD) PTIdi [0.064 (0.019)] and PTImus [0.065 (0.023)] were not different compared to infants without a history of mechanical ventilation [0.062 (0.019) and 0.065 (0.023), p = 0.574 and p = 0.841, respectively].

## Discussion

We have demonstrated that in convalescent infants, the invasively-derived pressure time index of the diaphragm and the non-invasive pressure–time index of the inspiratory muscles had a moderate degree of correlation and the non-invasive index could thus, potentially be used in clinical practice to assess inspiratory muscle function.

Assessment of inspiratory muscle function by measurement of a non-invasive pressure–time index of the respiratory muscles (PTImus) was first described by Gaultier et al. in children with chronic obstructive pulmonary disease [11]. Previous studies have also reported values of the PTIdi and PTImus in infants. Currie et al. measured the PTIdi and PTImus in twenty ventilated infants prior to extubation, with a median gestational age of 31 weeks and reported that a PTIdi of > 0.15 and a PTImus of > 0.18 were 100% sensitive and 100% specific in predicting extubation failure [14]. We have also previously measured fifty-six ventilated preterm infants and reported that a PTIdi of ≤ 0.12, and PTImus ≤ 0.10 were the most accurate predictors of extubation outcome with zero false-positive results [5]. Our study is the first to report values of both indices in convalescent infants prior to discharge, not receiving any respiratory support. In our study we report lower values of both indices corresponding to a lower work of breathing. Since the aforementioned studies reporting higher values of PTPdi and PTImus were performed in ventilated infants, the differences might be explained by the effect of mechanical ventilation which can compromise respiratory muscle performance [15]. The actual work of breathing would also be higher in ventilated infants during the acute phase of the disease, because of the pathology that necessitated the initiation of invasive ventilation and comorbidities such as previous systemic or respiratory infection [16]. Furthermore, the differences in our study compared to the previously mentioned studies might be explained by a different breathing pattern in more mature infants prior to discharge compared to the less mature, ventilated preterm infants of the previous studies.

We should note that, although at a population level we demonstrated a high level of agreement between the two methods, the scatter of the difference between the two methods was relatively large. It follows that there would be a group of infants that the non-invasive index could either over- or underestimate the actual diaphragmatic pressure time index. This difference, however, was only related to postmenstrual age and not to gestational age, birth weight and mechanical ventilation suggesting that the non-invasive index is reliable in infants born smaller or less mature with a diverging difference in the infants who were most mature at the time of assessment. In our population, a history of mechanical ventilation was not associated with impaired respiratory muscle function, the median duration of invasive ventilation, however, was only three days. The infants in our study were assessed prior to discharge and were by definition well, seemingly limiting the direct applicability of our results to well infants. We have previously measured the PTIdi and PTImus in ventilated infants and reported similarly that they are both meaningful in the clinical setting [5]. This study adds to the literature that respiratory muscle function assessment can also be undertaken by face mask in convalescent infants, and could possibly identify infants with severely abnormal respiratory muscle function who could potentially be at a greater risk of complications post discharge from neonatal care.

The non-invasive PTImus produced slightly higher values compared to the PTIdi. A possible explanation for this difference is that the PTImus corresponds to all respiratory muscles, while the PTIdi is based only on diaphragmatic measurements. Furthermore, the mean pressure is estimated in PTImus, but automatically calculated for PTIdi. The limitations of the PTImus to approximate the PTIdi have been previously described: in calculating the non-invasive mean inspiratory pressure, the rise in pressure during inspiration is approximated assuming a linear increase of the pressure. If, however, the inspiratory driving pressure increases exponentially with time, then the mean inspiratory pressure is over-estimated by extrapolating the occlusion pressure over the entire inspiratory time [17]. Another possible methodological consideration would be that the application of the face mask might alter the breathing pattern of unsedated infants [18] [19] affecting the ratio of inspiratory time to total time of respiration. The impact of the face mask though would be similar for both maximum pressure measurements and would theoretically not affect the relationship between the two indices.

Our study has strengths and some limitations. To our knowledge this is the first study to validate the non-invasive pressure–time index of the inspiratory muscles against the gold-standard PTIdi calculated by transdiaphragmatic pressure measurements. Furthermore, PTImus is a global respiratory muscle index, which does not overlook the possibly important effects of the accessory respiratory muscles on respiration. We used a large population of infants who were clinically well and were studied prior to discharge. This practically means that the term infants had only been admitted for a brief period of time but the preterm infants had spent enough time as inpatient to be at a late preterm/term corrected gestation when studied. We, thus, were not able to validate this index in very small or in premature infants in the early days of life. These infants, though are known to have limited muscle mass [20] and diaphragmatic functional reserves so, by definition, their inspiratory muscle function does not constitute a diagnostic conundrum. Finally, a long-term career break of the primary investigator did not permit for earlier publication of our data explaining the high rate of mechanically ventilated infants in our population.

In conclusion, in term and convalescent preterm infants, the non-invasive pressure–time index of the inspiratory muscles had a moderate degree of correlation with the invasively derived pressure time index of the diaphragm measured with a transdiaphragmatic catheter.
